# The Effect of Venture Capital on Enterprise Benefit According to the Heterogeneity of Human Capital of Entrepreneur

**DOI:** 10.3389/fpsyg.2020.01558

**Published:** 2020-07-10

**Authors:** Xin Jin, Puyang Zheng, Ziqi Zhong, Yali Cao

**Affiliations:** ^1^Business School of Guilin Tourism University, Guangxi Tourism Research Institute, Guilin, China; ^2^School of Economics and Management, East China Jiaotong University, Nanchang, China; ^3^Graduate School of Pan-Pacific International Studies, Kyung Hee University, Yongin-si, South Korea; ^4^Business School, Beijing Technology and Business University, Beijing, China

**Keywords:** VC of human capital, China VC database, enterprise innovation ability, enterprise benefits, Wind China financial database

## Abstract

This research was conducted for the purpose of exploring the role of venture capital (VC) based on the heterogeneity of human capital in the process of transforming innovation capabilities of enterprise into enterprise benefits and providing a reference for further research on enterprise performance development. In this study, 399 listed companies that obtained VC before 2018 from the China VC database and Wind China financial database were selected as research objects, and relevant data of patent number, return on equity, Tobin’s Q ratio, and research and development (R&D) investment ratio of each enterprise were obtained. Enterprise innovation ability was introduced to construct the relationship model of human-capital VC, enterprise innovation ability, and enterprise benefit, and the relationship between human-capital VC, enterprise innovation ability, and enterprise benefit was analyzed by the multiple-regression model. The results show that the number of patents of invested enterprises has an extremely significant positive correlation with the human capital index (*P* < 0.001), and a significant positive correlation with the education level of personnel, the proportion of engineering professionals (*P* < 0.05). The return on equity and Tobin’s Q ratio of enterprises have extremely significant positive correlations with the number of patents of invested enterprises (*P* < 0.001) and have a significant positive correlation with the number of patents of the invested enterprises × education level of the personnel, and the number of patents of the invested enterprises × proportion of engineering professionals (*P* < 0.05). All in all, the education level of the entrepreneur’ s VC human capital and the proportion of engineering professionals can effectively improve the innovation ability of the enterprise, thus indirectly playing a value-added role in the improvement of enterprise benefits. The invested enterprises will also face the problems of scale diseconomy and financing constraint when their profitability is enhanced, so they need to optimize their own business strategies.

## Introduction

Venture capital (VC) is mainly a financing method to provide financial support to startups and then acquire the shares of the company (Cheah and Ho, 2019; Lin et al., 2020). From the perspective of global industrial development, VC is an innovative capital, which can promote the innovation and economic progress of enterprises. Since the establishment of the first VC company in the United States in the 1950s, VC has played an important role in social and economic development (Del Bosco et al., 2019). It was in the mid to late 1990s that VC really developed an industry from a concept in China. Almost all successful technology companies that are famous in China, especially Internet companies, are basically supported by VC (Tiba et al., 2019), including Sina, Netease, Tencent, Baidu, and Alibaba. VC not only provides a certain amount of capital but also includes professional management experience, human resources, social relationship resources, and other aspects. The different resources have different influences on entrepreneurial enterprises. Among them, the human capital of an enterprise refers to the age, educational background, personal experience, and other aspects, and it is the behavior of making profits by investing in human capital (Liang et al., 2019; Owen and Mason, 2019; Wang et al., 2019). As an important human capital of economic development, entrepreneurs show multidimensional heterogeneity, which is divided into three parts: spirit heterogeneity, ability heterogeneity, and activity heterogeneity. Spiritual heterogeneity is mainly reflected in innovation, ability heterogeneity is mainly reflected in employing judgment, and activity heterogeneity is mainly reflected in entrepreneurship and management activities. Different types of human capital have different modes of action and contribution intensity to enterprise benefits (Capelleras et al., 2019; Assaker et al., 2020). As far as the amount of human capital of an enterprise is concerned, the population increases, the number of labor force increases, and the total human capital increases. As far as the quality of human capital is concerned, the knowledge and skills of human-capital owners lead to differences in labor productivity allocation. Therefore, the heterogeneity of human capital leads to significant differences in industrial innovation. Therefore, it is necessary to study human-capital venture investment based on entrepreneur heterogeneity during the economic development of enterprises.

The enterprise benefit refers to the proportion between the enterprise’s gross product and production cost, and it is also the fundamental purpose of VC. The relationship between VC and enterprise benefit has attracted the attention of many scholars (Gao et al., 2019; Ma et al., 2019). For example, Zhu (2019), based on the asymmetric information theory, expounded the behavior of VC from the perspectives of reputation, game model, VC, start-ups, and one-off return of numerous transactions and believed that the expected increase in the number of future transactions is the incentive for VC institutions to maintain a good reputation. Lei and Yarong (2019) studied the impact of industrial competition intensity between VC companies and invested enterprises on innovation input and output under the background of China by the random-effect model of unbalanced panel data based on the sample data of initial public offering (IPO) companies of the growth enterprise market (GEM) and small and medium-sized enterprises (SME) from 2008 to 2016. The results showed that by giving priority to investing in the adjacent start-ups and increasing the investment parent company’s participation in the management of the invested enterprises, the technology innovation output efficiency of the invested enterprises can be effectively improved and the technology innovation performance of the invested enterprises can also be optimized. Therefore, most previous researches focus on the whole enterprise or industry field, but there are few detailed unilateral analyses on the relationship between VC of human capital and enterprise performance (Heredia-Calzado and Duréndez, 2019). In this research, it intends to study the value-added effect of VC on enterprises from the perspective of human capital.

To sum up, although there are many researches on the relationship between VC of human capital of entrepreneur and enterprise performance, there are few targeted researches on the VC of human capital. Based on this, in this study, data of 399 listed companies from Shenzhen, Shanghai, and Beijing stock exchanges that obtained VC before 2018 from the China VC database and Wind China financial database were selected, and a relationship model of VC of human capital, enterprise innovation ability, and enterprise benefit was constructed. The relationship between human-capital VC, enterprise innovation ability, and enterprise benefit was analyzed by the multiple-regression model. Also, the role of VC based on human-capital heterogeneity in the transformation of enterprise innovation ability to enterprise benefit was comprehensively evaluated.

## Literature Review

Talent is the most important driving force of scientific and technological progress and an important pioneer of advanced productive forces. Human capital has a very significant impact on the long-term development of enterprises, which has also attracted the attention of many scholars. Brymer et al. (2019) elaborated on how enterprises strategically managed to channel human-investment portfolios and how they created inter-enterprise heterogeneity of human-capital resources, and proposed that different types of human capital had different impacts on the innovation development of enterprises. Sahasranamam and Nandakumar (2020), by determining the nature of the accidental influence of formal institutions on the relationship between personal capital and the emergence of social enterprises, found that personal capital had a positive effect on the benefits of social enterprises, which made a substantial contribution to the literature research on the promotion of social enterprise benefits. Huggins et al. (2017) investigated how theories related to human capital, growth drivers, and location conditions explained the survival and development of enterprises in a region and proposed that human capital related to entrepreneurs’ experience and growth drivers generated by strategic choices would affect the survival rate of enterprises. Some scholars also pay attention to the management and investment of human capital. Through a survey of Slovak enterprises, Kucharčíková and Mičiak (2018) found that increasing the value of human capital through the implementation of training can improve the efficiency, performance, competitiveness, and sustainability of transport enterprises. Song et al. (2019) used static and dynamic models to test the hypothesis and analyzed the large-scale data of China’s a-share-listed companies. The results showed that the human resource practice on the knowledge stock can improve the core competitiveness of the company, and the private VC type also had a great influence on the company performance. In addition, it was pointed out that the increase in the proportion of highly skilled manpower had a positive promoting effect on the investment of enterprise performance. Chanda and Goyal (2019) used the partial least-square method to examine the relationship between individual differences of employees and organizational performance. The results showed that investing a large amount of money in human capital to utilize employees’ productivity can effectively improve the performance of manufacturing organizations in India.

To sum up, the current studies on the effect of human-capital heterogeneity on enterprise benefits are limited to the input of enterprises themselves, and the influence of third-party investment institutions is ignored. Therefore, the influence of human-capital VC institutions on the invested enterprises is explored by building a model of the relationship between human capital index and enterprise performance, and innovation ability.

## Methodology

### Research Object

**FIGURE 1:**
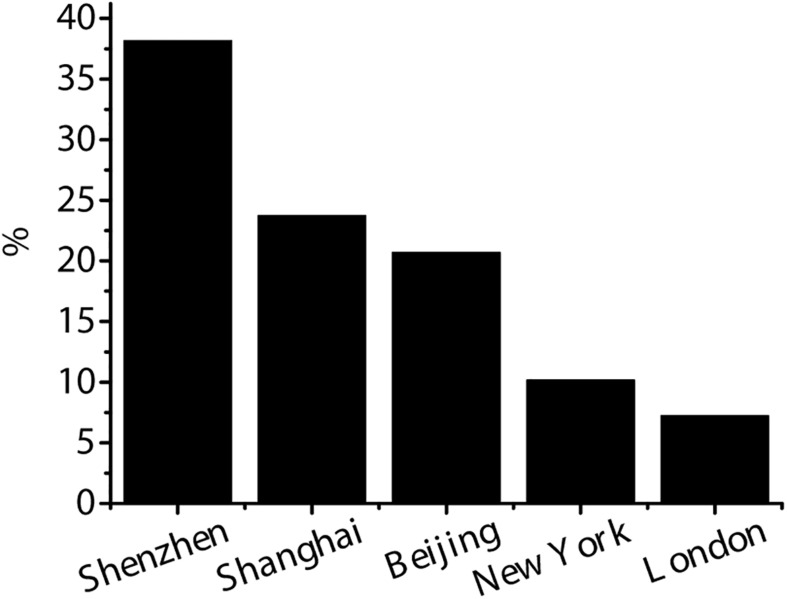
Distribution of the selected exchanges of listed companies.

The data of this study comes from the China VC database and Wind China financial database. 399 listed companies that obtained VC before 2018 were selected, including Shenzhen stock exchange, Shanghai stock exchange, Beijing stock exchange, New York stock exchange, and London stock exchange. The proportion of listed companies in various exchanges is shown in Figure 1. Shenzhen stock exchange has the largest number of listed companies, with 184, accounting for 38.16%. Shanghai stock exchange is second with 115, accounting for 23.74 %. There are 100 Beijing stock exchanges, accounting for 20.69%, 49 New York stock exchanges, accounting for 10.17%, and 35 London stock exchanges, accounting for 7.24%.

Due to the different requirements of different regional markets for the listed companies, the data lacks comparability. As can be observed from Figure 1, the listed companies in Shenzhen stock exchange, Shanghai stock exchange, and Beijing stock exchange account for 82.59% of the total number of companies. Therefore, considering the availability of financial data, the research scope is limited to 399 listed enterprises invested in Shenzhen, Shanghai, and Beijing stock exchanges.

**FIGURE 2:**
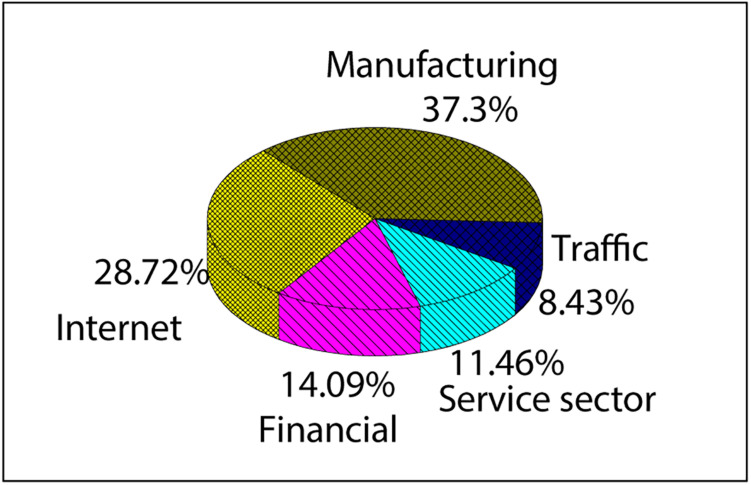
Proportion of industry distribution of 399 enterprises.

Figure 2 shows the industry distribution of 399 enterprises, including manufacturing industry, Internet industry, service industry, financial industry, and transportation industry. It can be observed that the invested enterprises are mainly distributed in the manufacturing and Internet industries, with a total of 149 manufacturing enterprises (37.30%) and 115 Internet enterprises (28.72%).

### China Venture Investment Database and Wind China Financial Database

The China venture investment database (Zhang and Passerini, 2020) is an online database that is easy to search and updated in real time. The data span from 2000 to the present. The main contents covered include more than 400 VC/private equity (PE) active in China, more than 5,000 related figures (including investors and entrepreneurs), more than 2,000 transaction data (including the transaction records of VC/PE investment, M&A, and listing), and more than 1,000 detailed information of the invested enterprises supported by VC/PE. All the data in this database are fed back from the questionnaire, and the database can be put into storage only after the second review by the researchers. This database can help users to realize a variety of requirements, including the analysis of industrial investment trends, looking for valuation and financial information, looking for secondary investment opportunities, and understanding the information of enterprise listing and mergers and acquisitions.

The Wind China financial database (Liu et al., 2020) is a leading enterprise in financial data, information, and software service in China. The financial terminal has a powerful Excel data link function, which is convenient for users to dynamically obtain real-time market situation, financial data, macro industry, and other data. Its covers the following (including all data from history to date and updated in real time): First, the transaction data, financial data, and all kinds of publicly disclosed information of major financial products in China’s securities market: stocks, funds, bonds, commodities and futures, foreign exchange, etc.; second, China stock market index: all basic information and trading data of Shanghai and Shenzhen stock exchange index, interbank bond market index, Xinhua FTSE index, China credit index, Shenyin Wanguo index, MSCI (Morgan Stanley Capital International), Dow Jones, and other indexes; third, Macro industry database: China’s macro economy, regional economy, overseas economy, industry, and press regulations; and fourth, featured databases including Hong Kong stocks, China’s overseas listed stocks, an earnings forecasts.

### Research Hypothesis

Assaker et al. (2020) proposed that human capital in a high-end catering industry had a structural impact on enterprise performance using response unit segmentation technology. Hamilton and Sodeman (2020) used big data of the HRM center to identify and develop knowledge stars that contributed too much to enterprise innovation, so as to help enhance enterprise capacity. Therefore, along with previous literature studies, it was concluded that human capital had dimensions to improve enterprise performance. In order to further explore the influence mechanism of human-capital characteristic VC on enterprise benefits, in this study, enterprise innovation ability is introduced as an intermediate variable. The human capital of an enterprise can be divided into three aspects: the educational level of the personnel, the proportion of the personnel who major in engineering, and the proportion of the personnel who have entrepreneurial experience.

Enterprise benefit can be divided into profitability and growth ability. Thus, the following assumptions can be made.

First is the relationship between human-capital VC and enterprise innovation ability.

S1: the VC level of human capital has a significant positive effect on the innovation ability of enterprises.
S1-1: the education level of investors has a significant positive effect on the innovation ability of enterprises.
S1-2: the proportion of investors that major in engineering has a significant positive effect on the innovation ability of enterprises.
S1-3: the proportion of investors with entrepreneurial experience has a significant positive effect on the innovation ability of enterprises.

Second is the relationship between enterprise innovation ability and enterprise benefit.

S2: enterprise innovation ability has a significant positive effect on enterprise benefit.
S2-1: the innovation ability of enterprises has a significant positive effect on the profitability of enterprises.
S2-2: the innovation ability of enterprises has a significant positive effect on the growth ability of enterprises.

Third is the relationship between VC of human capital and enterprise innovation ability and enterprise benefit.

S3: the higher the level of VC of human capital, the greater the impact of enterprise innovation ability on enterprise benefit.
S3-1: the higher the education level of the investors, the greater the impact of the enterprise’s innovation ability on the enterprise benefits.
S3-2: the higher the proportion of the investors who major in engineering, the greater the impact of the enterprise’s innovation ability on the enterprise benefits.
S3-3: the higher the proportion of the investors with entrepreneurial experience, the greater the impact of the enterprise’s innovation ability on the enterprise benefits.

### The Hypothesis Model of the Relationship Between Entrepreneurial Psychological Capital, Creative Innovation Behavior, and Enterprise Performance

#### Human-Capital VC and Enterprise Innovation Ability

At present, no mature system has been formed in the empirical research on human capital of VC institutions or the innovation ability of enterprises, which makes it difficult to build models. The number of patents of the technological innovation output index is selected to measure the innovation ability of enterprises. Human-capital VC is measured in three aspects: education level, proportion of engineering professionals, and proportion of people with entrepreneurial experience. Moreover, a multiple-regression model is constructed to test the effect of human capital and various factors of VC institutions on the innovation ability of enterprise.

(1) $$I\,n\,o\,v\,a\,t\,i\,o\,n_{i}=\beta_{1}\,C\,I\,R_{i,j}+\beta_{i}\,c\,o\,n\,t\,r\,o\,l\,V\,a\,r\,1$$

In Eq. 1, *Inovation*_*i*_ represents the innovation ability of the invested enterprise, *CIR*_*i,j*_ represents the human capital index of entrepreneur j’s investment in the invested enterprise, β_1_ and β_*i*_ are constant terms, and *controlVar1* represents the control variables, including the influencing factors of the enterprise (R&D investment, development stage) and risk financing factors (VC type and shareholding ratio).

Then, on the basis of Eq. 1, the relationship model between different dimensions of human-capital investment (education level of personnel, proportion of personnel in engineering major, proportion of personnel with entrepreneurial experience) and enterprise innovation ability is constructed.

(2) $$I\,n\,o\,v\,a\,t\,i\,o\,n_{i}=\beta_{1}\,e\,d\,u\,c\,a\,t\,i\,o\,n_{i,j}+\beta_{i}\,c\,o\,n\,t\,r\,o\,l\,V\,a\,r\,1$$

(3) $$I\,n\,o\,v\,a\,t\,i\,o\,n_{i}=\beta_{1}\,n\&e\,P\,e\,r_{i,j}+\beta_{i}\,c\,o\,n\,t\,r\,o\,l\,V\,a\,r\,1$$

(4) $$I\,n\,o\,v\,a\,t\,i\,o\,n_{i}=\beta_{1}\,e\,n\,t\,r\,e\,P\,e\,r_{i,j}+\beta_{i}\,c\,o\,n\,t\,r\,o\,l\,V\,a\,r\,1$$

In Eqs. 2–4, *education*_*i,j*_ represents the education level of VC personnel, *n*&*ePer*_*i*,*j*_ represents the proportion of engineering personnel, and *entrePer*_*i,j*_ represents the proportion of personnel with entrepreneurial experience. The above four models are set as S1, S11, S12, and S13, respectively.

Second is the enterprise innovation ability and enterprise benefit.

According to hypothesis S2 above, the following model is constructed:

(5) $$P\,e\,r\,f\,o\,r\,m\,a\,n\,c\,e_{i}=\beta_{1}\,I\,n\,o\,v\,a\,t\,i\,o\,n_{i}+\beta_{i}\,c\,o\,n\,t\,r\,o\,l\,V\,a\,r\,2$$

In Eq. 5, *Performance*_*i*_ represents enterprise benefit and *controlVar2* represents control variables, including R&D investment ratio, industry, and enterprise scale.

In this research, the enterprise benefits are divided into profitability and growth ability. Profitability is expressed by rate of return on common stockholders’ equity (ROE), and growth ability is expressed by Tobin’s Q ratio. Therefore, a more detailed model is built on the basis of model (5).

(6) $$R\,O\,E_{i}=\beta_{1}\,I\,n\,o\,v\,a\,t\,i\,o\,n_{i}+\beta_{i}\,c\,o\,n\,t\,r\,o\,l\,V\,a\,r\,2$$

(7) $$T\,o\,b\,i\,n^{\prime }\,s\,\,Q\,R\,a\,t\,i\,o_{i}=\beta_{1}\,I\,n\,o\,v\,a\,t\,i\,o\,n_{i}+\beta_{i}\,c\,o\,n\,t\,r\,o\,l\,V\,a\,r\,2$$

Eqs. 6 and 7, respectively represent the relationship model between enterprise innovation ability and enterprise profitability or growth ability.

Third are human-capital VC, enterprise innovation ability, and enterprise benefit.

To better verify the impact of VC of human capital on the benefit of the enterprise, the cross variables *Inovation*_*i*_ and *CIR*_*i,j*_ are used for multiple-regression modeling.

$$P\,e\,r\,f\,o\,r\,m\,a\,n\,c\,e_{i}=\beta_{1}\,I\,n\,o\,v\,a\,t\,i\,o\,n_{i}+\beta_{2}\,C\,I\,R_{i,j}$$

(8) $$+\beta_{3}\,I\,n\,o\,v\,a\,t\,i\,o\,n_{i}+C\,I\,R_{j}+\beta_{i}\,c\,o\,n\,t\,r\,o\,l\,V\,a\,r\,3$$

In Eq. 8, *controlVar3* represents the control variables, including the proportion of R&D investment, industry, development stage, enterprise scale, and year.

Then, on the basis of model (8), a new model was constructed to transform the enterprise’s innovation ability into the enterprise benefit from various dimensions of human-capital VC (education level of personnel, proportion of personnel major in engineering, and proportion of personnel with management experience).

$$P\,e\,r\,f\,o\,r\,m\,a\,n\,c\,e_{i}=\beta_{1}\,I\,n\,o\,v\,a\,t\,i\,o\,n_{i}+\beta_{2}\,e\,d\,u\,c\,a\,t\,i\,o\,n_{i,j}$$

(9) $$+\beta_{3}\,I\,n\,o\,v\,a\,t\,i\,o\,n_{i}\times e\,d\,u\,c\,a\,t\,i\,o\,n_{i,j}+\beta_{i}\,c\,o\,n\,t\,r\,o\,l\,V\,a\,r\,3$$

$$P\,e\,r\,f\,o\,r\,m\,a\,n\,c\,e_{i}=\beta_{1}\,I\,n\,o\,v\,a\,t\,i\,o\,n_{i}+\beta_{2}\,n\&e\,P\,e\,r_{i,j}$$

(10) $$+\beta_{3}\,I\,n\,o\,v\,a\,t\,i\,o\,n_{i}\times n\&e\,P\,e\,r_{i,j}+\beta_{i}\,c\,o\,n\,t\,r\,o\,l\,V\,a\,r\,3$$

$$P\,e\,r\,f\,o\,r\,m\,a\,n\,c\,e_{i}=\beta_{1}\,I\,n\,o\,v\,a\,t\,i\,o\,n_{i}+\beta_{2}\,e\,n\,t\,r\,e\,P\,e\,r_{i,j}$$

(11) $$+\beta_{3}\,I\,n\,o\,v\,a\,t\,i\,o\,n_{i}\times e\,n\,t\,r\,e\,P\,e\,r_{i,j}+\beta_{i}\,c\,o\,n\,t\,r\,o\,l\,V\,a\,r\,3$$

Equations 9–11 are the relationship models between each dimension of human-capital VC and enterprise innovation ability and enterprise benefit, respectively. β_1_, β_2_, β_3_, and β_*i*_ are constant terms of this model. To undertake correlation analysis, in this research, the number of patents *Patent*_*i*_ (technology innovation output index) is adopted to quantitatively represent the innovation ability of enterprises.

### Variable Calculation

Table 1 shows the introduction and calculation methods of independent variables, dependent variables, and control variables of each model.

**TABLE 1:** Introduction of each variable of the model.

**Variable name**	**Variable type**	**English symbols**	**Calculation method**
Total number of patents of the invested enterprise *i*	Dependent variable	*Patent*_*i*_	Including utility model patents, design patents, etc.
Enterprise benefit	Dependent variable	*Performance*_*i*_	Including net assets, Tobin’s Q ratio
Human capital index	Independent variable	*CIR*_*i,j*_	Including education level (0 for junior college and below, 1 for bachelor, and 2 for master and above), engineering background (1: yes, 0: no), entrepreneurial experience (1: yes, 0: no)
Educational level of personnel	Independent variable	*education*_*i,j*_	$e\,d\,u\,c\,a\,t\,i\,o\,n_{i,j}=\frac{1}{n}\,\sum_{k=0}^{n}e\,d\,u_{k}$, *n* indicates the number of members of human-capital VC, *edu*_*k*_ uses years of education as a weight
Proportion of engineering professionals	Independent variable	*n*&*ePer*_*i*,*j*_	$n\&e\,P\,e\,r_{i,j}=\frac{n\&e}{n}$, *n* indicates the number of members of human-capital VC, *n*&*e* indicates the number of engineering professionals
Proportion of people with entrepreneurial experience	Independent variable	*entrePer*_*i,j*_	$e\,n\,t\,r\,e\,P\,e\,r_{i,j}=\frac{e\,n\,t\,r\,e}{n}$, *n* indicates the number of members of human-capital VC, *entre* indicates the number of people with entrepreneurial experience
The proportion of enterprise R & D investment	Control variable	*R*&*D*_*i*_	*R*&*D*_*i*_ = *R*&*Dinvestment*_*i*_/*revenue*_*i*_,*R*&*Dinvestment*_*i*_ indicates the total amount of R & D investment funds, and *revenue*_*i*_ indicates the total revenue of the enterprise
The development stage of the enterprise	Control variable	*Stage*_*i*_	Dummy variables, including the early stage, development, maturity, and harvest (0, 1, 2, and 3)
Industry	Control variable	*industry*_*i*_	Dummy variable, which is classified by securities industry (the manufacturing industry is set to 4, the Internet is set to 3, the service industry is set to 2, the financial industry is set to 1, and the transportation industry is set to 0)
Years	Control variable	*Year*	Value based on 2005–2016
Enterprise scale	Control variable	*Size*_*i*_	*Size*_*i*_ = *log*⁡(*Asset*_*i*_), *Asset*_*i*_ represents the total assets of the enterprise
Type of VC	Control variable	*investType*_*i,j*_	Joint investment is set to 1, and the remaining types are set to 0
Shareholding ratio of investors	Control variable	*stockPer*_*i,j*_	Proportion of shares obtained by the investor in the invested enterprise

### Analytical Method

SPSS19.0 was used to process the data in this study. The relationship between human-capital VC investment and enterprise innovation ability, the relationship between enterprise innovation ability and enterprise benefit, and the role of human-capital VC investment in the process of transforming enterprise innovation ability into enterprise benefit were analyzed by multiple-regression model. Origin7.5 was used for plotting.

## Results

### Descriptive Statistics of Selected Enterprises

**FIGURE 3:**
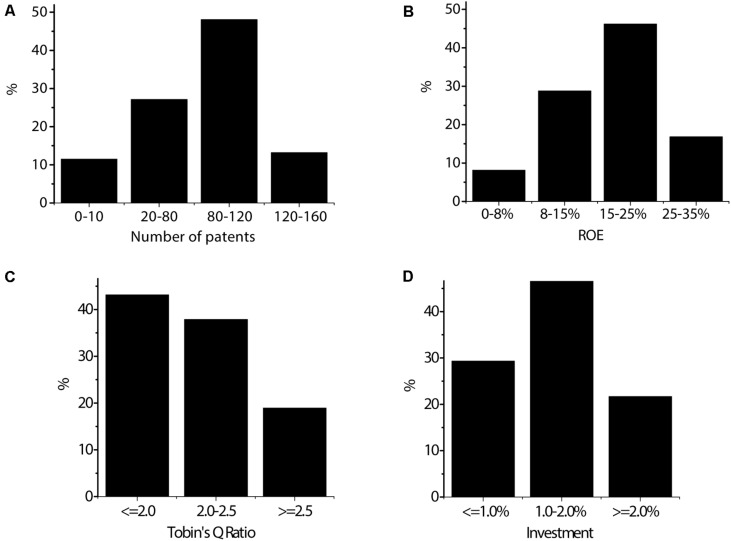
Distribution of the selected exchanges of listed enterprises. **(A)** The number of patents of the invested enterprise. **(B)** The return on equity of the invested enterprise. **(C)** The Tobin’s Q ratio of the invested enterprise. **(D)** The proportion of R&D investment of the invested enterprise.

As shown in Figure 3, there are 46 enterprises with 0–10 patents, accounting for 11.52%; 108 enterprises with 20–80 patents, accounting for 27.17%; 192 enterprises with 80–120 patents, accounting for 48.09%; and 53 enterprises with 120–160 patents, accounting for 13.22%. There are 33 enterprises with a return on equity of 0–8%, accounting for 8.16%; 115 enterprises with a return on equity of 8–15%, accounting for 28.81%; 184 enterprises with a return on equity of 15–25%, accounting for 46.19%; and 67 enterprises with a return on equity of 25–35%, accounting for 16.84%. There are 172 enterprises with a Tobin’s Q ratio less than 2.0, accounting for 43.17%; 151 enterprises with a Tobin’s Q ratio of 2.0–2.5, accounting for 37.88%; and 76 enterprises with a Tobin’s Q ratio greater than 2.5, accounting for 18.95%. There are 117 enterprises with an R&D investment ratio less than 1.0%, accounting for 29.37%; 195 enterprises with an R&D investment ratio of 1.0–2.0%, accounting for 48.92%; and 87 enterprises with an R&D investment ratio more than 2.0%, accounting for 21.71%.

### Descriptive Statistics of Variables

In this research, the variables of 399 enterprises in the model WERE sourced from the China venture investment database and Wind China financial database. The assignment method WAS adopted for some variables, and the descriptive statistics are shown in Table 2.

**TABLE 2:** Descriptive statistics of all variables in the model.

**Variable**	**Average value**	**Standard deviation**	**Maximum**	**Minimum**
Total number of patents of invested enterprise i	51.39	29.18	118	6
Education level of personnel	0.96	0.31	2	0
Proportion of engineering professionals	41.72%	18.11%	71.56%	15.37%
Proportion of people with entrepreneurial experience	32.06%	11.57%	56.78%	14.07%
Proportion of enterprise R&D investment	1.12%	0.92%	0.04%	3.68%
Types of VC	0.51	0.36	0	1
Shareholding ratio of investors	13.64%	8.29%	0.86%	43.57%
Enterprise development stage	1.72	1.36	4	0

### Empirical Research Results of Human-Capital VC and Enterprise Innovation Ability

As shown in Table 3, taking the number of patents of invested enterprises as the dependent variable, human capital index, personnel education level, proportion of engineering professionals, proportion of personnel with entrepreneurial experience, and four control variables (proportion of R&D investment, development stage, type of VC, and shareholding ratio of investors) as independent variables, the multifactor regression model analysis is conducted. It can be observed that the number of patents of invested enterprises has a very significant positive correlation with the human capital index (*P* < 0.001), and hypothesis S1 is true. There is a significant positive correlation between the number of patents of invested enterprises with the education level of the personnel, the proportion of engineering professionals, and the shareholding ratio of the investors (*P* < 0.05), and hypothesis S11 and hypothesis S12 are valid. There is no significant correlation between the number of patents of invested enterprise and the proportion of people with entrepreneurial experience, the proportion of R&D investment, the development stage of the enterprise, and the type of VC (*P* > 0.05), and hypothesis S13 is not true.

**TABLE 3:** Regression analysis of the number of patents of invested enterprises and innovation ability of enterprises.

**Model**		***t* value**	**Regression coefficients**	***P***
Independent variables	Human capital index	8.593	0.771**	0.000
	Educational level of personnel	5.617	0.712*	0.008
	Proportion of engineering professionals	5.860	0.603*	0.022
	Proportion of people with entrepreneurial experience	2.117	0.368	0.056
Control variables	The proportion of enterprise R&D investment	2.009	0.172	0.084
	Enterprise development stage	1.825	0.243	0.071
	Type of VC	1.338	0.308	0.086
	The shareholding ratio of the investors	4.610	0.518*	0.039

* *P* < 0.05; ** *P* < 0.001.

### The Empirical Research Results of Enterprise Innovation Ability and Enterprise Benefit

As shown in Table 4, the multifactor regression model analysis was carried out with enterprise return on equity as the dependent variable, the number of patents of invested enterprises, and the four control variables (industry of the enterprise, proportion of R&D investment, year, and enterprise scale) as the independent variables. There is an extremely positive correlation between the return on equity of enterprises and the number of patents of invested enterprises (*P* < 0.001), and hypothesis S21 is true. There is a significant negative correlation between the return on equity of enterprises, the proportion of R&D investment, and the size of enterprises (*P* < 0.05). Moreover, there is no significant correlation between the return on equity of the enterprise and the industry and year to which the enterprise belongs (*P* > 0.05).

**TABLE 4:** Regression analysis of return on equity and innovation ability of enterprises.

**Model**		***t* value**	**Regression coefficients**	***P***
Independent variables	Number of patents of invested enterprises	6.437	0.692**	0.000
Control variable	Industry	3.111	0.386	0.069
	Years	3.065	0.271	0.082
	The proportion of enterprise R&D investment	4.817	−0.588*	0.004
	Enterprise scale	4.289	−0.526*	0.006

* *P* < 0.05; ** *P* < 0.001.

As shown in Table 5, Tobin’s Q ratio is taken as the dependent variable, and the number of patents of invested enterprises and the four control variables (the industry of the enterprise, the proportion of R&D investment, the year, and the size of the enterprise) are used as the independent variables to undertake multifactor regression model analysis. It is found that the Tobin’s Q ratio of enterprises is positively correlated with the number of patents of invested enterprises (*P* < 0.001), and hypothesis S22 is true. There is a significant negative correlation between the Tobin’s Q ratio of enterprises, the proportion of enterprise R&D investment, and enterprise scale (*P* < 0.05). Moreover, there is no significant correlation between enterprise Tobin’s Q ratio and the industry and year of the enterprise (*P* > 0.05).

**TABLE 5:** Regression analysis of enterprise Tobin’s Q ratio and enterprise innovation ability.

**Model**		***t* value**	**Regression coefficients**	***P***
Independent variable	Number of patents of invested companies	7.551	0.763**	0.000
Control variable	Industry	1.458	0.205	0.087
	Years	1.197	0.183	0.052
	The proportion of enterprise R&D investment	4.066	−0.423*	0.011
	Enterprise scale	5.541	−0.517*	0.014

* *P* < 0.05; ** *P* < 0.001.

### Empirical Research Results on Human Capital VC, Enterprise Innovation Ability, and Enterprise Benefit

As shown in Table 6, the human-capital turnover item is introduced to study the role of human-capital VC in the transformation of enterprise’s innovation ability into enterprise benefit. Taking enterprise return on equity as the dependent variable, with the number of patents of the invested enterprise, number of patents of invested enterprises × human capital index, number of patents of invested enterprises × education level of personnel, number of patents of invested enterprises × proportion of engineering professionals, number of patents of the invested enterprises × the proportion of personnel with entrepreneurial experience, and seven control variables (the proportion of R&D investment, the stage of development, the type of VC, the shareholding ratio of investors, the industry, the year, and the scale of the enterprise) as independent variables, multiple-regression model analysis is conducted. It can be observed that there is a significant positive correlation between the return on equity of enterprises, the number of patents of invested enterprises, the number of patents of invested enterprises × human capital index, the number of patents of invested enterprises × education level of personnel, and the number of patents of invested enterprises × proportion of engineering professionals (*P* < 0.05), and hypotheses S3, S31, and S32 are true. There is a significant negative correlation between the return on equity of enterprises, the proportion of R&D investment, and the size of enterprises (*P* < 0.05). Also, there is no significant correlation between the return on equity of the enterprise with the number of patents of the invested enterprise × the proportion of people with entrepreneurial experience, the type of VC, the shareholding ratio of investors, the industry of the enterprise, and the year (*P* > 0.05), and hypothesis S33 is not true.

**TABLE 6:** The role of human-capital VC in transforming enterprise’s innovation ability into enterprise’s benefit (return on equity).

**Model**		***t* value**	**Regression coefficients**	***P***
Independent variable	Number of patents of invested enterprises	7.148	0.539*	0.005
	Number of patents of invested enterprises × human capital index	6.362	0.581*	0.012
	Number of patents of invested enterprises × education level of personnel	6.071	0.611*	0.043
	Number of patents of invested enterprises × proportion of engineering professionals	6.356	0.570*	0.031
	Number of patents of the invested company × proportion of personnel with entrepreneurial experience	4.961	0.226	0.068
Control variable	Proportion of R&D investment of enterprise	5.825	−0.543*	0.021
	Enterprise development stage	1.338	0.308	0.086
	Type of VC	2.529	0.178	0.050
	Shareholding ratio of investors	1.773	0.219	0.054
	Industry of the enterprise	2.909	0.316	0.085
	Year	4.776	0.255	0.063
	Enterprise scale	5.119	−0.631*	0.028

* *P* < 0.05.

In Table 7, multiple-regression model analysis was conducted with Tobin’s Q ratio as the dependent variable. It can be observed that there is a significant positive correlation between the Tobin’s Q ratio of enterprises, the number of patents of the invested enterprises, the number of patents of the invested enterprises × human capital index, the number of patents of the invested enterprises × the education level of the personnel, the number of patents of the invested enterprises × the proportion of engineering professionals, and the shareholding ratio of investors (*P* < 0.05). There is a significant negative correlation between the Tobin’s Q ratio of enterprise and enterprise size (*P* < 0.05). Also, there is no significant correlation between the Tobin’s Q ratio with the proportion of R&D investment, the development stage of the enterprise, the number of patents of the invested enterprise × the proportion of people with entrepreneurial experience, the industry of the enterprise, and the year of the enterprise (*P* > 0.05).

**TABLE 7:** The role of human-capital VC in transforming enterprise’s innovation ability into enterprise’s benefit (Tobin’s Q ratio).

**Model**		***t* value**	**Regression coefficients**	***P***
Independent variable	Number of patents of invested enterprises	7.148	0.539*	0.005
	Number of patents of invested enterprises × human capital index	6.362	0.581*	0.012
	Number of patents of invested enterprises × education level of personnel	6.071	0.611*	0.043
	Number of patents of invested enterprises × proportion of engineering professionals	6.356	0.570*	0.031
	Number of patents of the invested company × proportion of personnel with entrepreneurial experience	4.961	0.226	0.068
Control variable	Proportion of R & D investment of enterprise	5.825	−0.543*	0.021
	Enterprise development stage	1.338	0.308	0.086
	Type of VC	2.529	0.178	0.050
	Shareholding ratio of investors	1.773	0.219	0.054
	Industry of the enterprise	2.909	0.316	0.085
	Year	4.776	0.255	0.063
	Enterprise scale	5.119	−0.631*	0.028

* *P* < 0.05.

## Discussion

With the development of social economy, the transformation of economic model and industrial structure of all kinds of high-tech enterprises is imminent, and the state attaches more and more importance to the innovation ability of enterprises. As an incubation tool for SME and even large enterprises, VC can encourage enterprise innovation and improve the competitive advantage of enterprises through the support of external capital, manpower, and experience (Scarpellini et al., 2018; Schmutzler and Lorenz, 2018; Golovenkin et al., 2020). Therefore, in this study, the variable of enterprise innovation ability is firstly introduced to analyze the relationship between enterprise human-capital VC and enterprise innovation ability. The results show that the number of patents of invested enterprises has a very significant positive correlation with the human capital index (*P* < 0.001), which is consistent with the research results of Roper and Hewitt-Dundas (2017), indicating that the higher the human capital of VC, the stronger the innovation ability of enterprise, that is, the invested enterprises have gained innovative value through the process of human VC. However, there is no significant correlation between the number of patents and the proportion of R&D investment (*P* > 0.05), which further proves that the appreciation of enterprises’ innovation ability does not come from the invested enterprises themselves but from investors. By the education years method, it is found that the number of patents of invested enterprises has a significant positive correlation with the education level of employees (*P* < 0.05), which indicates that the education level of investors has a positive impact on the innovation ability of enterprises. Besides, there is a significant positive correlation between the number of patents of invested enterprises and the proportion of engineering professionals and the shareholding ratio of investors (*P* < 0.05), which is the same as the field research results of Bianchini et al. (2017) on some SMEs, indicating that engineering professionals pay more attention to the actual R&D and technological innovation of enterprises and can provide professional value-added services, thus improving the innovation ability of enterprises. There are two explanations for the added value role of investors’ human VC. First, the investors provide technique, equity, and management advice to the invested enterprises with their own ability. Second, the investors provide cooperative help to the invested enterprises through their huge relationship resources. From the above analysis, it can be concluded that the added value effect of human-capital investment is more inclined to the ability possessed by investors, while social relationship resources and entrepreneurial experience are not reflected.

From the perspective of hypothetical selection, venture investors pay the most attention to the future development income of the enterprise, so the enterprise that can acquire investors has a promising development potential (Asad et al., 2018). In this study, it is found that there is a very significant positive correlation between the enterprise return on equity, Tobin’s Q ratio, and the number of patents of invested enterprise (*P* < 0.001), which is basically the same as the research results of Carayannis et al. (2017), indicating that the innovation ability of enterprises can be effectively converted into enterprise benefits, and the stronger the innovation ability, the better the profitability and growth ability of enterprises. In terms of control variables, there is a significant negative correlation between enterprise return on equity, Tobin’s Q ratio, the proportion of R&D investment, and enterprise scale (*P* < 0.05); the larger the scale of the enterprise, the higher the proportion of R&D investment, and the lower the benefit of the enterprise. This may be due to the fact that although the investors are optimistic about the development prospect of the enterprise and increase the investment in the enterprise, which makes the enterprise scale expand, many enterprises are still in the early stage of development with low profitability, and the increase in operating costs leads to the reduction in the enterprise benefits (Gu et al., 2018). In the same way, the investment in R&D will also increase the operating costs of enterprises, thus reducing the benefits of enterprises.

To better analyze the role of human-capital VC in the transformation of enterprise’s innovation ability into enterprise’s benefit, in this study, the human-capital crossover term is intentionally introduced. It is found that there is a significant positive correlation between the enterprise return on equity, the Tobin’s Q ratio, and the number of patents of invested enterprise, the number of patents of invested enterprise × the human capital index, the number of patents of invested enterprise × the education level of the personnel, and the number of patents of invested enterprise × the proportion of engineering professionals (*P* < 0.05), which is consistent with the research results of Vickers et al. (2017). Therefore, it can be concluded that in the process of transforming enterprise innovation abilities into enterprise benefits, the personnel education level and the proportion of personnel with engineering background all play a significant positive promoting role. Moreover, there is still a significant negative correlation between the enterprise return on equity, the Tobin’s Q ratio, the R&D investment of enterprise, and the size of the enterprise (*P* < 0.05) for the same reasons as above. Besides, there is no significant correlation between enterprise return on equity, the Tobin’s Q ratio, and the number of patents of invested enterprises × the proportion of people with entrepreneurial experience (*P* > 0.05), which is consistent with the research results of Babenko et al. (2017), indicating that the proportion of people with entrepreneurial experience has no significant effect on the transformation of enterprise’s innovation ability into enterprise benefit (Qian et al., 2018; Chen, 2019; Wu et al., 2019).

## Conclusion

In this study, the enterprise innovation ability was introduced to build the relationship model of human-capital risk investment, enterprise innovation ability, and enterprise benefit and analyzes the role of human-capital risk investment in the process of transforming enterprise innovation abilities into enterprise benefits, so as to provide reference for further research on the development of enterprise performance (Zhou and Wu, 2018). However, the development time of VC in China is short, the database related to VC is not complete, and the sample size of enterprises is small. The accuracy and availability of investment data can be analyzed in the future. All in all, the education level of human capital of entrepreneurs’ VC and the proportion of engineering personnel can effectively improve the innovation ability of enterprises, thereby indirectly playing an added value role in the improvement of enterprise benefits (Wu and Wu, 2019). The invested enterprises will also face the problems of scale diseconomy and financing constraint when their profitability is enhanced, so they need to optimize their own business strategies.

## Data Availability Statement

The raw data supporting the conclusions of this article will be made available by the authors, without undue reservation.

## Ethics Statement

The studies involving human participants were reviewed and approved by the Guilin Tourism University Ethics Committee. The patients/participants provided their written informed consent to participate in this study.

## Author Contributions

XJ: conceptualization, methodology, and writing – original draft preparation. PZ and ZZ: formal analysis and visualization. YC: writing – review and editing. All authors contributed to the article and approved the submitted version.

## Conflict of Interest

The authors declare that the research was conducted in the absence of any commercial or financial relationships that could be construed as a potential conflict of interest.

## References

[B1] Asad M. Shabbir M. Salman R. Haider S. Ahmad I. (2018). Do entrepreneurial orientation and size of enterprise influence the performance of micro and small enterprises? A study on mediating role of innovation. *Manag. Sci. Lett.* 8 1015–1026. 10.5267/j.msl.2018.7.008

[B2] Assaker G. Hallak R. O’Connor P. (2020). Examining heterogeneity through response-based unit segmentation in PLS-SEM: a study of human capital and firm performance in upscale restaurants. *Curr. Issues Tour.* 23 137–152. 10.1080/13683500.2018.1490253

[B3] Babenko V. Romanenkov Y. Yakymova L. Nakisko O. (2017). Development of the model of minimax adaptive management of innovative processes at an enterprise with consideration of risks. *BocmoЧchno*-Eponeŭckuŭ yuurnal nepeδoeblx mexhoЛosuŭ 5 49–56. 10.15587/1729-4061.2017.112076

[B4] Bianchini A. Donini F. Pellegrini M. Saccani C. (2017). An innovative methodology for measuring the effective implementation of an Occupational Health and Safety Management System in the European Union. *Saf Sci.* 92 26–33. 10.1016/j.ssci.2016.09.012

[B5] Brymer R. A. Chadwick C. Hill A. D. Molloy J. C. (2019). Pipelines and their portfolios: a more holistic view of human capital heterogeneity via firm-wide employee sourcing. *Acad. Manag. Perspect.* 33 207–233. 10.5465/amp.2016.0071

[B6] Capelleras J. L. Contin-Pilart I. Larraza-Kintana M. Martin-Sanchez V. (2019). Entrepreneurs’ human capital and growth aspirations: the moderating role of regional entrepreneurial culture. *Small Bus. Econ.* 52 3–25. 10.1007/s11187-017-9985-0

[B7] Carayannis E. G. Meissner D. Edelkina A. (2017). Targeted innovation policy and practice intelligence (TIP2E): concepts and implications for theory, policy and practice. *J. Technol. Transf.* 42 460–484. 10.1007/s10961-015-9433-8

[B8] Chanda U. Goyal P. (2019). Assessing impact of human capital, srhrm and employee related factors on firm performance. *J. Indust. Integr. Manag.* 04 72–105.

[B9] Cheah S. Ho Y. P. (2019). Building the ecosystem for social entrepreneurship: University Social Enterprise Cases in Singapore. *Sci. Technol. Soc.* 24 507–526. 10.1177/0971721819873190

[B10] Chen M. (2019). The impact of expatriates’ cross-cultural adjustment on work stress and job involvement in the high-tech industry. *Front. Psychol.* 10:2228. 10.3389/fpsyg.2019.02228 31649581 PMC6794360

[B11] Del Bosco B. Chierici R. Mazzucchelli A. (2019). Fostering entrepreneurship: an innovative business model to link innovation and new venture creation. *Rev. Manag. Sci.* 13 561–574. 10.1007/s11846-018-0318-8

[B12] Gao J. Schøtt T. Sun X. Liu Y. (2019). Heterogeneous effects of business collaboration on innovation in small enterprises: china compared to Brazil, Indonesia, Nigeria, and Thailand. *Emerg. Mark. Finance Trade.* 55 795–808. 10.1080/1540496x.2018.1510310

[B13] Golovenkin E. N. Vilkov V. Y. Matronitsky D. A. Voroshilova A. S. (2020). Analytical approach to the diversification effect evaluation of the innovative engineering projects portfolio for a machine-building enterprise//IOP Conference Series: materials Science and Engineering. *IOP Publ* 734:012037 10.1088/1757-899x/734/1/012037

[B14] Gu W. Saaty T. L. Wei L. (2018). Evaluating and optimizing technological innovation efficiency of industrial enterprises based on both data and judgments. *Int. J. Inform. Technol. Decis. Making* 17 9–43. 10.1142/s0219622017500390

[B15] Hamilton R. H. Sodeman W. A. (2020). The questions we ask: opportunities and challenges for using big data analytics to strategically manage human capital resources. *Bus Horiz.* 63 85–95. 10.1016/j.bushor.2019.10.001

[B16] Heredia-Calzado M. Duréndez A. (2019). The influence of knowledge management and professionalization on the use of ERP systems and its effect on the competitive advantages of SMEs. *Enterp. Inf. Syst.* 13 1245–1274. 10.1080/17517575.2019.1640393

[B17] Huggins R. Prokop D. Thompson P. (2017). Entrepreneurship and the determinants of firm survival within regions: human capital, growth motivation and locational conditions. *Entrepreneursh. Reg. Dev.* 29 357–389. 10.1080/08985626.2016.1271830

[B18] Kucharčíková A. Mičiak M. (2018). Human capital management in transport enterprises with the acceptance of sustainable development in the Slovak Republic. *Sustainability* 10:2530 10.3390/su10072530

[B19] Lei W. Yarong Q. (2019). The competition intensity of CVC and the innovation performance of invested enterprises: an analysis of the mediating effect of involvement intensity. *J. Shanghai Univ. Finance Econ.* 21 46–64.

[B20] Liang C. Ip C. Y. Wu S. C. Law K. M. Y. Wang J. H. Peng L. P. (2019). Personality traits, social capital, and entrepreneurial creativity: comparing green socioentrepreneurial intentions across Taiwan and Hong Kong. *Stud. High. Educ.* 44 1086–1102. 10.1080/03075079.2017.1418310

[B21] Lin R. Xie Z. Hao Y. Wang J. (2020). Improving high-tech enterprise innovation in big data environment: a combinative view of internal and external governance. *Int. J. Inf. Manag.* 50 575–585. 10.1016/j.ijinfomgt.2018.11.009

[B22] Liu T. Qu S. Scherpereel C. M. (2020). The influence of the role positioning of investment institutions on the value of start-up enterprises from the perspective of network. *Sustainability* 12:491 10.3390/su12020491

[B23] Ma H. Sun Q. Gao Y. Gao Y. (2019). Resource integration, reconfiguration, and sustainable competitive advantages: the differences between traditional and emerging industries. *Sustainability* 11:551 10.3390/su11020551

[B24] Owen R. Mason C. (2019). Emerging trends in government venture capital policies in smaller peripheral economies: lessons from Finland, New Zealand, and Estonia. *Strateg. Chang.* 28 83–93. 10.1002/jsc.2248

[B25] Qian J. Song B. Jin Z. Wang B. Chen H. (2018). Linking empowering leadership to task performance, taking charge, and voice: the mediating role of feedback-seeking. *Front. Psychol.* 9:2025. 10.3389/fpsyg.2018.02025 30410461 PMC6209672

[B26] Roper S. Hewitt-Dundas N. (2017). Investigating a neglected part of Schumpeter’s creative army: what drives new-to-the-market innovation in micro-enterprises? *Small Bus. Econ.* 49 559–577. 10.1007/s11187-017-9844-z

[B27] Sahasranamam S. Nandakumar M. K. (2020). Individual capital and social entrepreneurship: role of formal institutions. *J. Bus. Res.* 107 104–117. 10.1016/j.jbusres.2018.09.005

[B28] Scarpellini S. Marín-Vinuesa L. M. Portillo-Tarragona P. Moneva J. M. (2018). Defining and measuring different dimensions of financial resources for business eco-innovation and the influence of the firms’ capabilities. *J. Clean. Prod.* 204 258–269. 10.1016/j.jclepro.2018.08.320

[B29] Schmutzler J. Lorenz E. (2018). Tolerance, agglomeration and enterprise innovation performance: a multilevel analysis of Latin American regions. *Ind. Corp. Chang.* 27 243–268.

[B30] Song M. Pan X. Pan X. Jiao Z. (2019). Influence of basic research investment on corporate performance. *Manag. Decis.* 57 1839–1856.

[B31] Tiba S. van Rijnsoever F. J. Hekkert M. P. (2019). Firms with benefits: a systematic review of responsible entrepreneurship and corporate social responsibility literature. *Corp. Soc. Responsib. Environ. Manag.* 26 265–284.

[B32] Vickers I. Lyon F. Sepulveda L. McMullin C. (2017). Public service innovation and multiple institutional logics: the case of hybrid social enterprise providers of health and wellbeing. *Res. Policy* 46 1755–1768.

[B33] Wang L. Zhou F. An Y. Yang J. (2019). Corporate venture capital: technological innovation or value creation? A comparative study of CVC-and IVC-invested Chinese listed companies. *Asian J. Technol. Innov.* 27 257–279.

[B34] Wu T. J. Wu Y. J. (2019). Innovative work behaviors, employee engagement, and surface acting. *Manag. Decis.* 57 3200–3216.

[B35] Wu W. Wang H. Zheng C. Wu Y. J. (2019). Effect of Narcissism, Psychopathy, and Machiavellianism on Entrepreneurial Intention—The Mediating of Entrepreneurial Self-Efficacy”. *Front. Psychol.* 10:360. 10.3389/fpsyg.2019.00360 30846958 PMC6393355

[B36] Zhang M. Passerini K. (2020). From fan-centric handset manufacturing to intense product diversification: a study of the rapid transformation of a telecommunication Giant in China and Beyond. *Int. J. Interdiscip. Telecommun. Netw.* 12 22–33.

[B37] Zhou F. Wu Y. J. (2018). How humble leadership fosters employee innovation behaviour. *Leadersh. Organ. Dev. J.* 39 375–387.

[B38] Zhu Q. (2019). The relationship between reputation and behavior of venture capital institutions under the theory of information asymmetry. *Am. J. Indus. Bus. Manag.* 9 315–324.

